# Identifying the research priorities of healthcare professionals in UK vascular surgery: modified Delphi approach

**DOI:** 10.1093/bjsopen/zraa025

**Published:** 2020-12-28

**Authors:** G E Smith, J Long, T Wallace, D Carradice, I C Chetter, Vascular Research Collaborative, L Atkin, D Beard, M Bown, A Bradbury, A Cook, P Coughlin, A Handa, R Hinchliffe, C Imray, M Jenkins, R Lathan, J McCaslin, B Modarai, T Rashid, T Richards, C Rogers, D Russell, R Sayers, L Sharples, D Sidloff, R Simpson, G Stansby, P Stather, D Torgerson, S R Vallabhaneni

## Abstract

**Background:**

The Vascular Research Collaborative was established to develop a national research strategy for patients with vascular disease in the UK. This project aimed to establish national research priorities in this patient group.

**Methods:**

A modified Delphi approach, an established method for reaching a consensus opinion among a group of experts in a particular field, was used to survey national multidisciplinary vascular clinical specialists. Two rounds of online surveys were conducted involving the membership of the Vascular Society, Society of Vascular Nurses, Society for Vascular Technology, and the Rouleaux Club (vascular surgical trainees). The first round invited any suggestions for vascular research topics. A steering group then collated and rationalized the suggestions, categorizing them by consensus into pathological topics and research categories, and amalgamating the various questions relating to the same fundamental issue into a single question. The second round involved recirculating these questions to the same participants for priority scoring.

**Results:**

Round 1 resulted in 1231 suggested research questions from 481 respondents. Steering group collation and rationalization resulted in 83 questions for ranking in round 2. The second round resulted in a hierarchical list of vascular research priorities. The highest scoring priorities addressed topics related to critical lower-limb ischaemia, diabetic foot disease, amputation, wound healing, carotid plaque morphology, and service organization/delivery.

**Conclusion:**

It is anticipated that these results will drive the UK national vascular research agenda for the next 5–10 years. It will facilitate focused development and funding of new research projects in current clinical areas of unmet need where potential impact is greatest.

## Introduction

Vascular patients present with a wide range of disease processes in the arterial, venous and lymphatic systems, frequently requiring the involvement of multidisciplinary specialist teams. This work frequently overlaps with other specialties to treat patients with common and debilitating conditions, such as diabetes, stroke, renal failure, cardiac disease and trauma. The number of patients under the care of vascular specialists is growing rapidly, as is the array and complexity of investigations and interventions employed in diagnosing and managing vascular disease.

Although there is already a highly active academic vascular surgery community in the UK, with over 40 per cent of the consultant workforce in academic roles^1^, there is currently no agreement regarding research priorities within the specialty. Indeed, academic vascular surgery units are frequently in direct competition for the limited available research funds.

Vascular surgery became an independent surgical specialty in the UK in 2013, and in 2016 the Vascular Research Collaborative (VRC) was established, with a remit of developing a national strategy for vascular research. As its first project, the VRC aimed to establish a list of clinical research questions, formulated and prioritized by the current vascular workforce. The VRC considered this process to be valuable to both academic vascular clinicians and to research funding bodies. Vascular academics would have a prioritized national work list, which would strengthen funding applications and promote a collaborative approach to vascular clinical trials. Funding bodies would have greater confidence in awarding limited resources to trials directly answering high-priority questions in areas of current equipoise, with a clear direct impact on patient care.

The modified Delphi method applies a series of structured surveys to reach a consensus regarding a complex problem. This method has been used to identify research priorities in other specialties, and is particularly helpful where significant differences of opinion or high levels of uncertainty exist^2^^,^^3^. A central steering panel manages the Delphi process, organizing the initial survey, collating and rationalizing the initial survey results, before redistribution for reconsideration by the wider group of experts. This process may occur several times to clarify the position of the wider group. The method therefore benefits from the ongoing involvement of the wider expert group whilst concurrently directing opinion towards a clear consensus. Additionally, the method generates clear documentation by which final consensus was reached^4^.

The aim of this study was to produce a prioritized list of questions to guide and direct future vascular surgical research.

## Methods

The steering group, convened from the members of the VRC and chaired by the surgical specialty lead for vascular surgery, included representatives from societies being surveyed and regional Clinical Research Network leads for vascular surgery. Invitation to participate as part of the wider expert group was extended to all members of relevant societies whose committees had agreed to be involved.

### Round 1

The steering group produced a paper version of the survey, which was assessed for clarity and face validity at the Vascular Society’s annual scientific meeting in November 2016. In January 2017, personalized links to the survey (*Appendix S1*) using an online survey tool (Bristol Online Survey, University of Bristol, Bristol, UK) were distributed directly to e-mail addresses to track responses. The e-mail addresses were supplied from the membership databases of the Vascular Society of Great Britain and Ireland, the Rouleaux Club, the Society of Vascular Nurses, and the Society for Vascular Technology. These national UK societies represent a large number of clinicians responsible for the management of patients with vascular disease. An open link was also included in an invitation circulated via global e-mails for forwarding by recipients, so that relevant specialists who were not members of the societies might also be able to respond. The survey collected brief demographic data and details of research interests and experience. Respondents then suggested as many research questions for prioritization in free text as they wished. Non-responders received reminder e-mails after 2 and 4 weeks, and the first round closed in March 2017.

All round 1 responses were analysed and coded independently by two steering group members into pathology categories and topics (*Table 1*). Disagreements between the two assessors were resolved by discussion with a third. Responses that were nonsensical or could not be formulated into a research question were excluded, and responses addressing the same central problem were amalgamated. Finally, the whole steering group reviewed and rationalized this revised list of responses, agreeing a list of research questions, grouped according to pathological category, for prioritization in round 2.

**Table 1:** Pathology and topic categories rationalized after round 1 responses

Pathology category	Topic category
Amputations	Basic science and natural history
Aorta	Education (patient)
Blue toe syndrome	Education and training (staff)
Carotids	Endovascular management
Diabetic foot	Exclusions
Lymphoedema	Intervention
Mesenteric ischaemia	Investigations
Other	Non-surgical
Pseudoaneurysm	Miscellaneous
PAD (general arterial, iliac disease)	Open surgical management
Thoracic outlet	Outcomes and cost-effectiveness
Trauma	Perioperative care
Vascular access	Prevention
Vasculitis	Risk assessment/prediction
Vasospastic disorders	Screening
Venous (IVC filters, deep venous disease)	Service provision
Visceral aneurysms	Surveillance
Wound management (leg ulcers)	

PAD, peripheral arterial disease; IVC, inferior vena cava.

### Round 2

In August 2017, the rationalized, categorized list of research questions was recirculated to all invited participants from round 1 and respondents to the open link, who were asked to rank each question between 1 (least important) and 10 (most important). Non-responders received reminder e-mails at 2 and 4 weeks. This second (ranking) round closed in October 2017.

For analysis of round 2, multiple methods of analysis were used to interrogate the results, including the overall sum of scores received, mean scores received, number of top scores received, and a cluster group analysis. Questions were ranked for both importance overall and relative importance within each category. Steering group members convened again after closure of the second round to review the results of round 2 and decide whether a further round of ranking was necessary.

## Results

The committees of the Vascular Society of Great Britain and Ireland, the Society for Vascular Technology, the Society of Vascular Nurses and the Rouleaux Club of vascular trainees all agreed to their membership being contacted for potential participation in the Delphi process. Response rates were approximately 30 per cent in both survey rounds (*Table 2*).

**Table 2:** Survey response rates by specialty

	Survey round 1			Survey round 2	
**Specialty area**	**No. of surveys sent**	**No. of responses**	**Research questions submitted**	**No. of surveys sent**	**No. of responses**
Surgeons	983	281 (28.6)	829	585^*^	206 (35.2)
Nurses	115	81 (70.4)	172	115	36 (31.3)
Technologists	479	119 (24.8)	230	479	81 (16.9)
Total	1577	481 (30.5)	1231	1179	1 (27.4)

Values in parentheses are percentages.

*Reduction in no. of surveys sent due to updated membership list.

**Fig. 1:**
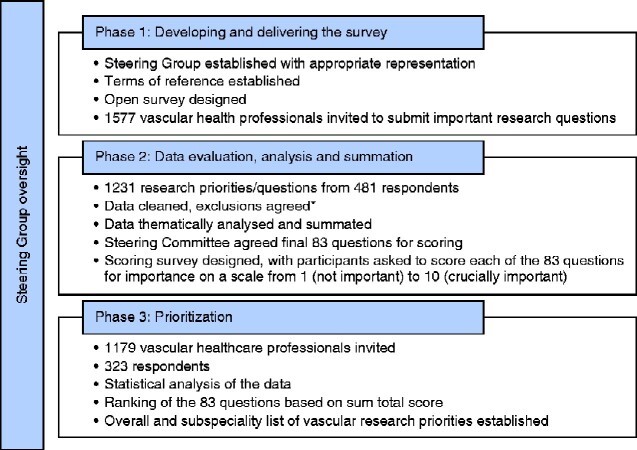
Overview of the modified Delphi process to prioritize research questions for the vascular research community *Reasons for reductions and exclusions included no identifiable question (for example statements), questions specific to an individual or consolidated into a common question.

Following validation, 1577 individuals were invited by e-mail to participate in round 1 of the survey, resulting in 481 responses suggesting 1231 research questions for prioritization. At least one response was received from over 90 per cent of units registered with the National Vascular Registry as active vascular centres in 2016. An overview of the Delphi process is shown in *Fig. 1*.

**Fig. 2:**
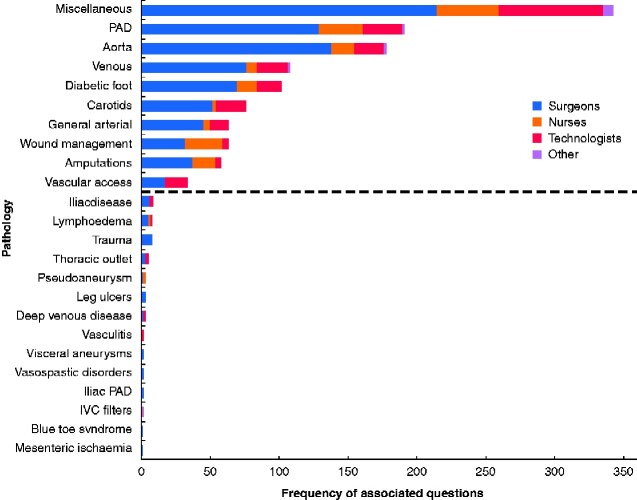
Frequency of submitted questions organized by pathology The dashed line denotes categories that received less than 1 per cent of all responses and were not taken forward to the ranking process in round 2. PAD, peripheral arterial disease; IVC, inferior vena cava.

The steering group agreed that, given the very large numbers of suggested questions received in round 1, a general rationalization was required based on overall response rates. Therefore, categories that received less than 1 per cent of all responses were considered to have demonstrated a low level of research priority and were not taken forward to the ranking process in round 2 (*Fig. 2*). This was felt necessary in order to make the round 2 survey manageable and not dissuade participation by presenting a survey that appeared too onerous and time-consuming. Questions labelled miscellaneous were further categorized or redistributed into existing categories, following review by the steering group. A final list of 83 questions in 10 pathology categories were produced for ranking in round 2, following consideration and agreement by the steering committee. By the closing date, 323 responses to round 2 had been received, with all questions ranked.

Steering group members convened again to review the analysis of round 2 results. All potential methods of data interrogation and question ranking returned similar results, with the majority of the top 10 ranking questions being consistent between all methods. Statistical advice recommended a simple sum of all scores received by each question as the most transparent method by which to rank the research questions. An overall league table of research priorities was produced, including ranked questions in each category (*Table 3*; for the full version, see *Appendix S2*).

**Table 3:** Top research priorities according to category (for full overall league table, see *Appendix S2*)

Ranking	Research question	Total score
	**Wound management**	
6^*^	What is the most effective way to manage mixed aetiology/hard to heal/complex leg ulcers?	2598
10^*^	Can we optimize wound healing in vascular patients?	2511
12^†^	How can we reduce surgical-site infection in vascular surgery?	2482
	**Diabetic foot**	
2^*^	What is the optimal revascularization strategy in diabetic patients?	2686
4^*^	How can we improve outcomes in diabetic patients with foot sepsis?	2634
16^†^	How can access to multidisciplinary diabetic foot care be improved?	2468
30^†^	How can we promote awareness of diabetic foot complications?	2380
	**Peripheral arterial disease**	
1^*^	What can be done to improve outcomes in critical limb ischaemia (including how best to identify those who would benefit from revascularization and those who would be best managed with primary amputation or palliation)?	2708
17^†^	How can we reduce progression of arterial disease?	2450
19^†^	Can we develop a critical limb ischaemia care pathway to ensure optimal management?	2442
	**Carotid**	
7^*^	Can we characterize carotid plaque to identify patients at high risk of events and target interventions?	2582
14^†^	What is the optimal management of patients with carotid disease using individualized risk benefit ratios?	2480
	**Vascular access**	
25^†^	How do we optimize patency rates following arteriovenous fistulas/grafts?	2414
	**Aorta**	
15^†^	What is the best treatment option for ‘complex’ AAA (e.g. short necks, juxtarenal, iliac pathologies)?	2469
20^†^	What is the optimal management of patients with aortic aneurysm disease using individualized risk : benefit ratios?	2441
23^†^	How do we improve long-term outcomes following EVAR?	2424
26^†^	What is the optimal post-EVAR surveillance strategy following endovascular AAA repair?	2413
28^†^	What is the optimal medical therapy for patients with AAA to minimize expansion/rupture?	2399
	**Amputation**	
3^*^	How can we reduce the rates of major lower limb amputations?	2638
5^*^	How can we improve clinical outcomes for patients following major limb amputation?	2623
18^†^	How can we optimize rehabilitation following major lower-limb amputation?	2449
27^†^	How can we optimize pain management (including phantom pain) following major lower-limb amputation?	2401
	General	
21^†^	How can we effectively prevent/slow progression of arteriosclerosis?	2437
22^†^	How can we optimize preoperative risk assessment and improve fitness in vascular patients?	2435
	**Venous**	
11^†^	What is the optimal treatment strategy for proximal deep venous disease (thrombolysis, stenting, compression, surgery, anticoagulation)?	2502
24^†^	Can we develop a leg ulcer care pathway to ensure optimal management?	2417
29^†^	Does early intervention in superficial venous incompetence prevent disease progression to ulceration?	2389
	**Service organization, access and delivery**	
8^*^	How can we best organize regional vascular services to facilitate optimal management and outcomes for vascular patients?	2533
9^*^	How do we optimize delivery of vascular services to improve patient experience and outcomes?	2514
	**Education and training**	
13^†^	How can we improve the vascular surgical curriculum to ensure high levels of competence in both open and endovascular surgery?	2481

*Ranked as a top 10 priority;

^†^ranked as a top 30 priority. There were no top 30 research priorities in Imaging or Research categories.

## Discussion

This modified Delphi method produced a prioritized list of research questions that reflect the clinical matters of greatest importance when treating patients with vascular disease. This was the first attempt to engage a wide group of frontline, multidisciplinary, healthcare experts to identify vascular research priorities that may have an impact on daily clinical practice. The modified Delphi process may be criticized for using small, academic ‘expert panels’, considered potential sources of bias. The use of a large, varied, predominantly non-academic ‘expert panel’ in this study is a notable strength, directing national research focus away from individual areas of specific interests towards the areas of daily practice most in need of a robust evidence base. The Delphi process used in this study was an efficient and transparent process to aggregate opinions effectively and to lead to a consensus within a large group of professionals^5^^,^^6^. Similar methods have been employed successfully in other surgical specialties, producing research priorities over a wide range of topics^2^^,^^7–9^. This priority list should direct research towards the greatest needs of vascular patients and assist funding bodies in developing themed calls. The relatively high response rates, which approached 30 per cent in both rounds, were significantly higher than observed in previous similar studies, and may be attributable to the extensive promotion of the study within the vascular healthcare community. Over 90 per cent of UK National Vascular Registry-registered vascular centres participated, suggesting excellent process engagement and valid representation of the views of the national vascular healthcare professional workforce. Furthermore, the analysis of responses stratified according to participant affiliation demonstrated broad agreement for each society within the overall list of priorities.

The top priority questions relate to common clinical dilemmas, where the current evidence base is sparse or conflicting. The highest priority question clearly reflects a frequent clinical conundrum of exposing elderly co-morbid patients to extensive surgery with a potentially significant mortality and failure rate. Subsequent questions cover a range of pathologies, although diabetic vascular care, major limb amputations and centralized service delivery all feature twice in the top 10. The prevalence of diabetes continues to increase, and presents specific problems in vascular care related to difficulties with perfusion assessments and, frequently, very distal and calcified disease. This increase in diabetes-related workload seems to be reflected in this research priority list. Conversely, the two priorities related to amputations are not as easily explained as the prevalence of major lower-limb amputations has fallen over time^10^^,^^11^. However, it has been highlighted that adherence to quality improvement frameworks and outcomes following major lower-limb amputation could be improved^12^. Service provision, appearing twice in the top 10 research priorities, is clearly a concern for the current vascular workforce. UK vascular services have recently been restructured to a more centralized ‘hub and spoke’ model, based mainly on evidence of improved outcomes from higher-volume centres^13^. However, in a specialty in which at least half of the workload relates to emergency or urgent care, the effects of centralization (such as delays in interhospital transfers) on patient experience should certainly be examined.

There are limitations to this Delphi research priority-setting process. Ideally, the steering group would not have excluded any valid round 1 questions from being recirculated in round 2, the prioritization stage. This was deliberated by the steering group and considered essential given the large volume of initial research questions generated in round 1, and the need to maintain a manageable burden in round 2. Every effort was made to ensure equal representation of the views of all vascular healthcare professionals, but response rates varied between societies. Whilst high numbers of surgeons and technologists participated, the response rate from specialist nurses (over 70 per cent in round 1) was approximately twice that of the other groups.

This priority list reflects the opinions of vascular healthcare professionals, but does not include input from service users (patients and carers). Despite this, organization of services to maximize outcomes and patient experience ranked highly in this research priority-setting process. This omission is currently being addressed via a James Lind Alliance patient and public priority-setting partnership to ensure the broadest possible involvement of stakeholders in vascular care^7^.

The findings of this study have the potential to develop a wide-reaching, international footprint by highlighting areas in the care of vascular patients in greatest need of underpinning research. Funding bodies are urged to consider promotion of these research priorities. Societies with members involved in the care of vascular patients are encouraged to champion these priorities and contribute to further development of research questions and funding applications. These results will set the agenda for research in vascular patients for the foreseeable future, promoting the development, funding and delivery of new studies in areas of utmost need where potential impact is greatest.

## Funding

Circulation Foundation provided expenses for steering group meetings and basic costs associated with the work.

## Collaborators

Other members of the Vascular Research Collaborative: L. Atkin, D. Beard, M. Bown, A. Bradbury, A. Cook, P. Coughlin, A. Handa, R. Hinchliffe, C. Imray, M. Jenkins, R. Lathan, J. McCaslin, B. Modarai, T. Rashid, T. Richards, C. Rogers, D. Russell, R. Sayers, L. Sharples, D. Sidloff, R. Simpson, G. Stansby, P. Stather, D. Torgerson and S. R. Vallabhaneni.

## Supplementary Material

zraa025_Supplementary_Data

## Notes

Other members of the Vascular Research Collaborative are co-authors of this study and are listed under the heading Collaborators

Presented to the Vascular Societies’ Annual Scientific Meeting, Manchester, UK, November 2017
