# Supplementary material for: Identifying the research priorities of healthcare professionals in UK vascular surgery: modified Delphi approach
Source: BJS Open. 2020 Dec 28;5(2):zraa025. doi: 10.1093/bjsopen/zraa025 (PMC7944495; doi:10.1093/bjsopen/zraa025)
Supplement: zraa025_Supplementary_Data [file zraa025_supplementary_data.zip › Supporting information 1.docx]

The Vascular Research Collaborative

Leanne  Atkin

David Beard

Mathew Bown

Andrew Bradbury

Andrew Cook

Patrick Coughlin

Ashok Handa

Rob Hinchliffe

Chris Imray

Michael Jenkins

Ross Lathan

James McCaslin

Bijan Modarai

Tawqeer Rashid

Toby Richards

Chris Rogers

David Russell

Rob Sayers

Linda Sharples

David Sidloff

Richard Simpson

Gerard Stansby

Philip Stather

David Torgerson

S.R.Vallabhaneni
