# Supplementary material for: Identifying the research priorities of healthcare professionals in UK vascular surgery: modified Delphi approach
Source: BJS Open. 2020 Dec 28;5(2):zraa025. doi: 10.1093/bjsopen/zraa025 (PMC7944495; doi:10.1093/bjsopen/zraa025)
Supplement: zraa025_Supplementary_Data [file zraa025_supplementary_data.zip › Appendix S1.docx]

**Appendix S1** Survey for vascular research

**A National Survey for Vascular Research**

Thank you for joining this important survey, your responses will directly inform the future Vascular Society Research Strategy. Remember you don’t have to be a researcher to get involved and we want to hear from anyone involved with the speciality. All views are welcome.

**First Name:**

**Last Name:**

**Email Address:**

**Associated Vascular Unit** (hospital name/location)**:**

| **Gender**  🞎Male  🞎Female |  |
| --- | --- |
| **Age**  🞎20-30 years  🞎31-40 years 🞎41-50 years | 🞎51-60 years  🞎61-70 years  🞎71+years |

**What is your vascular specialty?**

| 🞎 Vascular Surgery  🞎 Vascular Nursing  🞎 Vascular Technology  🞎 Vascular Radiology  🞎 Other |  |
| --- | --- |

If you selected Other, please specify:

**Do you have a particular area of interest? You can select more than one.**

| 🞎Abdominal Aortic Aneurysm  🞎Amputation  🞎Carotid Vascular Disease  🞎Claudication  🞎Clinical Trials  🞎Diabetes  🞎New technologies /registries  🞎Patient Public Involvement  🞎Peripheral Arterial Disease | 🞎Service improvement / development  🞎Systematic Reviews  🞎Surgical Education  🞎Ulcers  🞎Ultrasound  🞎Varicose veins  🞎Vascular access  🞎Wound Management  🞎Other |
| --- | --- |

If you selected Other, please specify:

**Involvement with Research**

1. **What best describes your involvement with vascular based research?**

🞎I am actively involved with vascular based research.

***Answer Q2.***

🞎I have been involved with vascular research in the past but not at present.

***Answer Q3.***

🞎I have never been involved with vascular research.

***Answer Q4.***

🞎I have never been involved with vascular research but I am interested.

***Answer Q4.***

1. **What best describes your current role in vascular research?**

| 🞎Chief Investigator  🞎Principal Investigator  🞎Sub Investigator | 🞎Research Nurse  🞎Trials Coordinator  🞎Other study staff |
| --- | --- |

If you selected Other, please specify:

**3a. What best describes your previous role in vascular research?**

| 🞎Chief Investigator  🞎Principal Investigator  🞎Sub Investigator | 🞎Research Nurse  🞎Trials Coordinator  🞎Other study staff |
| --- | --- |

If you selected Other, please specify:

**3b. What best describes the reason why you are no longer involved with vascular research?**

| 🞎Not interested  🞎Not enough time  🞎Not enough funding | 🞎Not enough staff  🞎Lack of facilities  🞎Other |
| --- | --- |

If you selected Other, please specify:

1. **What best describes the reason why you have never been involved with vascular research?**

| 🞎Not interested  🞎Not enough time  🞎Not enough funding | 🞎Not enough staff  🞎Lack of facilities  🞎Other |
| --- | --- |

If you selected Other, please specify:

**What are the future vascular research priorities?**

Please try to formulate your research topic into a question, for example;

- How do we improve access to services?
- How do you improve outcomes for major limb amputees?

You may include the above examples in your responses if you think they are important. Remember this can be about any topic e.g. clinical, basic science or service improvement.

Thank you for taking the time to complete this survey.
