# Supplementary material for: Identifying the research priorities of healthcare professionals in UK vascular surgery: modified Delphi approach
Source: BJS Open. 2020 Dec 28;5(2):zraa025. doi: 10.1093/bjsopen/zraa025 (PMC7944495; doi:10.1093/bjsopen/zraa025)
Supplement: zraa025_Supplementary_Data [file zraa025_supplementary_data.zip › Appendix S2.docx]

**Appendix S2** Overall league table of research priorities according to category (full version of *Table 3*)

|  | **Ranked as a top 10 priority** | |  | **Ranked as a top 30 priority** |
| --- | --- | --- | --- | --- |
|  | | | | |
| **Ranking** | | **Research Questions** | | **Total Score** |
|  | | **WOUND MANAGEMENT** | |  |
| **6** | | **What is the most effective way to manage mixed aetiology / hard to heal / complex leg ulcers?** | | 2598 |
| **10** | | **Can we optimise wound healing in vascular patients?** | | 2511 |
| **12** | | **How can we reduce Surgical Site Infection in vascular surgery?** | | 2482 |
|  | |  | |  |
|  | | **DIABETIC FOOT** | |  |
| **2** | | **What is the optimal revascularisation strategy in diabetic patients?** | | 2686 |
| **4** | | **How can we improve outcomes in diabetic patients with foot sepsis?** | | 2634 |
| **16** | | **How can access to multi-disciplinarily diabetic foot care be improved?** | | 2468 |
| **30** | | **How can we promote awareness of diabetic foot complications?** | | 2380 |
| 37 | | Can we improve risk assessment in patients with diabetic foot complications? | | 2307 |
| 75 | | Is screening for a diabetic foot pathology worthwhile? | | 2069 |
|  | |  | |  |
|  | | **PAD** | |  |
| **1** | | **What can be done to improve outcomes in critical limb ischaemia (including how best to identify those who would benefit from revascularisation and those who would be best managed with primary amputation or palliation)?** | | 2708 |
| **17** | | **How can we reduce progression of arterial disease?** | | 2450 |
| **19** | | **Can we develop a critical limb ischemia (CLI) care pathway to ensure optimal management?** | | 2442 |
| 34 | | What is the optimal assessment of distal vasculature and perfusion? | | 2318 |
| 36 | | What is the best medical therapy for PAD? | | 2311 |
| 38 | | How can we reduce cardiovascular risk in PAD patients? | | 2306 |
| 39 | | What is the optimal antiplatelet therapy following lower limb revascularisation? | | 2298 |
| 40 | | Is post revascularisation surveillance worthwhile and what is the optimal strategy (modality, timing)? | | 2298 |
| 47 | | How can we improve provision and access to exercise programmes for claudicants? | | 2266 |
| 51 | | What novel non invasive interventions are effective for claudication? | | 2224 |
| 63 | | Would a more aggressive revascularisation strategy improve outcomes in claudicants? | | 2148 |
| 65 | | How can we promote awareness of PAD? | | 2137 |
| 67 | | What is the optimal exercise prescription for claudication? | | 2122 |
| 70 | | Is pre-conditioning prior to PAD surgery feasible and effective? | | 2107 |
| 73 | | Is cell / gene therapy effective in PAD? | | 2092 |
| 78 | | Can decision trees and pathways of care be used to improve care for PAD patients? | | 1999 |
| 81 | | Is screening for PAD beneficial? | | 1906 |
|  | |  | |  |
|  | | **CAROTID** | |  |
| **7** | | **Can we characterise carotid plaque to identify patients at high risk of events and target interventions?** | | 2582 |
| **14** | | **What is the optimal management of patients with carotid disease using individualised risk benefit ratios?** | | 2480 |
| 43 | | What is the optimal antiplatelet regime following carotid endarterectomy? | | 2287 |
| 50 | | Is there an association between carotid disease and cognitive decline? | | 2237 |
| 82 | | Is enhanced recovery beneficial following carotid endarterectomy? | | 1903 |
| 83 | | What is the role of TCD in carotid surgery? | | 1872 |
|  | |  | |  |
|  | | **VASCULAR ACCESS** | |  |
| **25** | | **How do we optimise patency rates following arterio-venous fistulae / grafts?** | | 2414 |
| 48 | | Does post-operative surveillance improve patency rates of arteriovenous fistulae? | | 2256 |
|  | |  | |  |
|  | | **AORTA** | |  |
| **15** | | **What is the best treatment option for "complex" AAA (e.g. short necks, juxta renal, iliac pathologies)** | | 2469 |
| **20** | | **What is the optimal management of patients with aortic aneurysm disease using individualised risk benefit ratios?** | | 2441 |
| **23** | | **How do we improve long term outcomes following EVAR?** | | 2424 |
| **26** | | **What is the optimal post-EVAR surveillance strategy following endovascular AA repair?** | | 2413 |
| **28** | | **What is the optimum medical therapy for patients with AAA to minimise expansion / rupture?** | | 2399 |
| 31 | | What is the best management strategy for Type B aortic dissection? | | 2354 |
| 32 | | What is the optimal management of aortic graft infection? | | 2348 |
| 49 | | How can we improve our understanding of AAA biology in relation to promotion and growth? | | 2256 |
| 52 | | What are the most appropriate outcome measures in patients with AAA? | | 2215 |
| 56 | | How do we prevent spinal cord ischaemia during aortic aneurysm repair? | | 2188 |
| 58 | | What is the optimal pathway for patients undergoing AAA repair? | | 2182 |
| 68 | | What is the optimum AAA screening strategy? | | 2119 |
| 69 | | Should EVAR 2 be repeated in the modern era? | | 2118 |
| 74 | | What is the optimal surveillance strategy for "sub-threshold" aortas? | | 2081 |
| 79 | | Should EVAR 1 be repeated in the modern era? | | 1967 |
|  | |  | |  |
|  | | **AMPUTATION** | |  |
| **3** | | **How can we reduce the rates of major lower limb amputations?** | | 2638 |
| **5** | | **How can we improve clinical outcomes for patients following major limb amputation?** | | 2623 |
| **18** | | **How can we optimize rehabilitation following major lower limb amputation?** | | 2449 |
| **27** | | **How can we optimize pain management (inc phantom pain) following major lower limb amputation?** | | 2401 |
| 61 | | Which is better – above or through knee amputation? | | 2169 |
|  | |  | |  |
|  | | **GENERAL** | |  |
| **21** | | **How can we effectively prevent /slow progression of arteriosclerosis?** | | 2437 |
| **22** | | **How can we optimise pre-op risk assessment and improve fitness in vascular patients?** | | 2435 |
| 41 | | New and emerging technologies; how are they introduced and evaluated? | | 2293 |
| 42 | | Can we improve vascular surgical data collection, analysis, utilisation and reporting? | | 2290 |
| 45 | | How can we reduce length of stay for vascular patients? | | 2280 |
| 53 | | Can we develop a core outcome set for vascular procedures? | | 2212 |
| 55 | | What is the role of hybrid procedures for intervention in vascular surgery? | | 2198 |
| 60 | | How can we maximise awareness of cardiovascular disease and health to the wider healthcare teams and our patients? | | 2175 |
|  | |  | |  |
|  | | **VENOUS** | |  |
| **11** | | **What is the optimal treatment strategy for proximal deep venous disease (thrombolysis, stenting, compression, surgery, anti-coagulation)?** | | 2502 |
| **24** | | **Can we develop a leg ulcer care pathway to ensure optimal management?** | | 2417 |
| **29** | | **Does early intervention in superficial venous incompetence prevent disease progression to ulceration?** | | 2389 |
| 57 | | What is the optimal compression strategy (bandages, stockings, boots) for patients with venous disease and how do we improve compliance? | | 2188 |
| 59 | | What is the optimal VTE thromboprophylaxis strategy in VV intervention? | | 2182 |
| 62 | | What is the optimal commissioning policy for superficial venous incompetence? | | 2158 |
| 64 | | What is the optimal strategy for the diagnosis and management of calf DVT? | | 2138 |
| 66 | | How can we improve VTE prevention? | | 2131 |
| 71 | | Are non-thermal as effective as thermal ablative techniques in the management of varicose veins? | | 2107 |
| 72 | | How can the long term outcomes be improved following treatment for varicose veins? | | 2098 |
| 76 | | How prevalent is pelvic vein incompetence and is treatment effective? | | 2039 |
| 77 | | What is the optimal compression regime following endovenous ablation? | | 2015 |
| 80 | | What are the basic mechanisms underlying venous incompetence? | | 1956 |
|  | |  | |  |
|  | | **IMAGING** | |  |
| 54 | | What is the role of 3D and contrast enhanced ultrasound in vascular imaging? | | 2208 |
|  | |  | |  |
|  | | **SERVICE ORGANISATION, ACCESS AND DELIVERY** | |  |
| **8** | | **How can we best organise regional vascular services to facilitate optimal management and outcomes for vascular patients?** | | 2533 |
| **9** | | **How do we optimise delivery of vascular services to improve patient experience and outcomes?** | | 2514 |
| 35 | | How do we improve access to vascular services? | | 2311 |
|  | |  | |  |
|  | | **RESEARCH** | |  |
| 33 | | What can we do to promote research and improve recruitment to vascular clinical trials? | | 2321 |
| 44 | | How do we develop / evaluate the role of the vascular nurse specialist / practitioner? | | 2281 |
|  | |  | |  |
|  | | **EDUCATION AND TRAINING** | |  |
| **13** | | **How can we improve the vascular surgical curriculum to ensure high levels of competence in both open and endovascular surgery?** | | 2481 |
| 46 | | How do we maximise patient participation in vascular service evaluation and research? | | 2269 |
